# Analysis and Visualization Tool for Targeted Amplicon Bisulfite Sequencing on Ion Torrent Sequencers

**DOI:** 10.1371/journal.pone.0160227

**Published:** 2016-07-28

**Authors:** Stephan Pabinger, Karina Ernst, Walter Pulverer, Rainer Kallmeyer, Ana M. Valdes, Sarah Metrustry, Denis Katic, Angelo Nuzzo, Albert Kriegner, Klemens Vierlinger, Andreas Weinhaeusel

**Affiliations:** 1 Health & Environment Department, Molecular Diagnostics, AIT–Austrian Institute of Technology, Vienna, Austria; 2 The Department of Twin Research & Genetic Epidemiology, King’s College London, St Thomas’ Campus, London, United Kingdom; 3 Platomics GmbH, Vienna, Austria; UCLA-DOE Institute for Genomics and Proteomics, UNITED STATES

## Abstract

Targeted sequencing of PCR amplicons generated from bisulfite deaminated DNA is a flexible, cost-effective way to study methylation of a sample at single CpG resolution and perform subsequent multi-target, multi-sample comparisons. Currently, no platform specific protocol, support, or analysis solution is provided to perform targeted bisulfite sequencing on a Personal Genome Machine (PGM). Here, we present a novel tool, called TABSAT, for analyzing targeted bisulfite sequencing data generated on Ion Torrent sequencers. The workflow starts with raw sequencing data, performs quality assessment, and uses a tailored version of Bismark to map the reads to a reference genome. The pipeline visualizes results as lollipop plots and is able to deduce specific methylation-patterns present in a sample. The obtained profiles are then summarized and compared between samples. In order to assess the performance of the targeted bisulfite sequencing workflow, 48 samples were used to generate 53 different Bisulfite-Sequencing PCR amplicons from each sample, resulting in 2,544 amplicon targets. We obtained a mean coverage of 282X using 1,196,822 aligned reads. Next, we compared the sequencing results of these targets to the methylation level of the corresponding sites on an Illumina 450k methylation chip. The calculated average Pearson correlation coefficient of 0.91 confirms the sequencing results with one of the industry-leading CpG methylation platforms and shows that targeted amplicon bisulfite sequencing provides an accurate and cost-efficient method for DNA methylation studies, e.g., to provide platform-independent confirmation of Illumina Infinium 450k methylation data. TABSAT offers a novel way to analyze data generated by Ion Torrent instruments and can also be used with data from the Illumina MiSeq platform. It can be easily accessed via the Platomics platform, which offers a web-based graphical user interface along with sample and parameter storage. TABSAT is freely available under a GNU General Public License version 3.0 (GPLv3) at https://github.com/tadkeys/tabsat/ and http://demo.platomics.com/.

## Introduction

DNA methylation is one of the most important epigenetic modifications of the eukaryotic genome and plays essential roles in several biological processes, such as alternative splicing [1], regulation of temporal and spatial gene expression [2,3], as well as genome stabilization [4]. It is the most widely studied epigenetic modification in humans and describes the adding of a methyl group to DNA nucleotides, in humans typically occurring in a CpG dinucleotide context [5]. These CpG sites are often clustered together as CpG islands, which co-locate to distinct gene regions, modifying the expression of these genes. DNA methylation is heritable and known to cause genomic imprinting [6]. It is highly relevant for the study of human disease, as somatic alterations of the methylation status have been described to be associated with aging [7], atherosclerosis [8], and several human diseases [9], most notably cancer [10].

The gold-standard for studying single-base methylation is bisulfite sequencing, also referred to as BS-Seq [11]. Here, DNA is treated with bisulfite, which changes the DNA sequence depending on the methylation status of individual cytosine residues. Unmethylated cytosines are converted to uracil, which is amplified and read by the sequencer as thymine, whereas methylated cytosines are left unconverted. Next, the generated sequences are compared to a known reference yielding single nucleotide resolution information about the methylation status of the DNA. In cases where whole bisulfite genome sequencing is too expensive or not required, a practical alternative is to limit the sequencing per individual to selected, meaningful targets. This approach, commonly termed as targeted sequencing, allows generating consistent data with high coverage around regions of interest [12].

Due to the low entry cost and medium throughput, bench-top next-generation DNA sequencers (454 GS Junior from Roche, Illumina MiSeq, or Ion Torrent Personal Genome Machine (PGM)) are especially equipped for targeted sequencing [13]. The PGM [14] applies a sequencing-by-synthesis approach, uses native dNTP chemistry, and relies on a modified silicon chip to detect hydrogen ions released during base incorporation by DNA polymerase. The sequencer generates single-end (SE) reads in varying quality and length. A known caveat of the PGM is its susceptibility to over-call or under-call the number of homopolymer bases, a feature which needs to be specifically addressed by dedicated downstream analysis methods and tools [15].

Bisulfite sequencing data analysis involves several steps including quality assessment, alignment, and methylation calling [16]. Several tools are available, which use different approaches to analyze the data [17,18]. An important part of the bisulfite sequencing workflow is the translation of raw sequence information into bisulfite calls for each investigated base. The widely used tool Bismark [19] contains multiple routines to carry out alignment of bisulfite-treated reads to a reference genome as well as cytosine methylation calling.

According to omicsmaps.com there are over 300 Ion Torrent PGM machines in use, which could potentially be applied to targeted bisulfite sequencing. However, currently no protocol or analysis solution for bisulfite sequencing on the PGM is officially provided. We have therefore created a novel tool called TABSAT for the analysis and visualization of targeted bisulfite sequencing data generated by Ion Torrent instruments. The tool accepts raw sequencing files as input and outputs result tables containing information about the methylation status of covered CpG sites. Read mapping and methylation calling is handled by Bismark, which has been modified to use the TMAP [20] mapper instead of the default mapper Bowtie2. Results are aggregated in tabular format and automatically visualized as lollipop figures. TABSAT has been designed to run with a minimal set of input parameters but can be customized to support specific questions. In addition, it can be used with data from the Illumina MiSeq platform. The software is freely available at https://github.com/tadkeys/tabsat/.

## Overview of TABSAT

**Fig 1:**
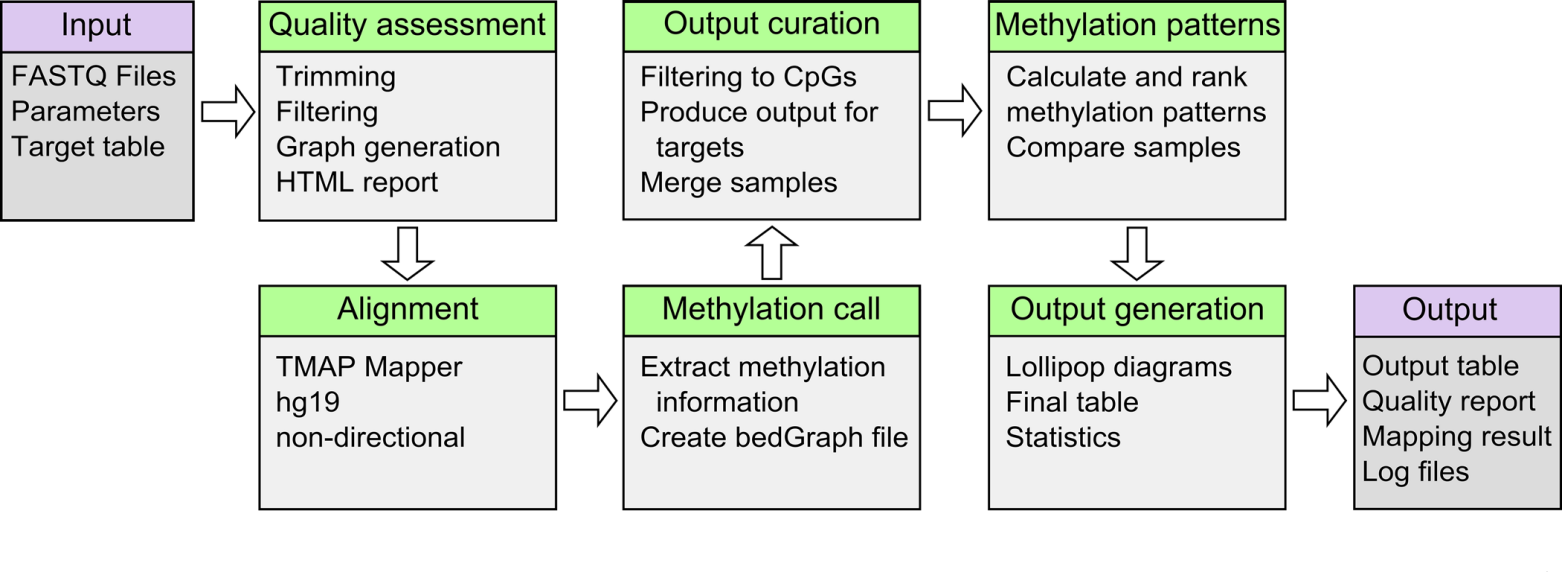
The analysis workflow of TABSAT is shown. The first step conducts quality assessment and generation of a report. Next, sequences are mapped to a reference genome and the methylation information is extracted. Based on several quality statistics and thresholds the generated results are filtered and aggregated into a final output table. In the next step, reads covering all CpGs in a target region are used to calculate methylation-pattern statistics, which are subsequently compared between samples. The last step creates the final output table, graphical representations of the results, and reports basic statistics generated during the analysis workflow.

**Fig 2:**
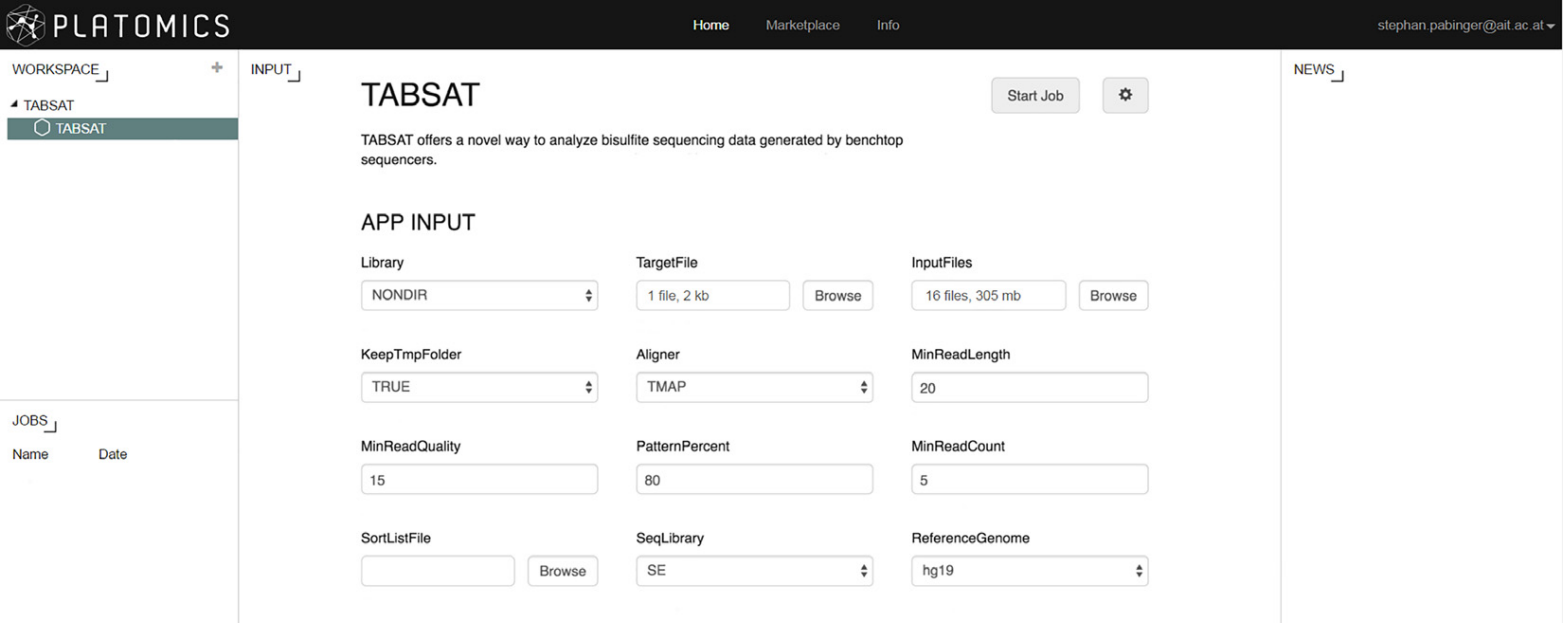
The Platomics input page of TABSAT is displayed. After setting the correct parameters, a new analysis is started by clicking on the “Start Job” button. Running and finished analyses are displayed in the jobs panel and can be selected by clicking on the corresponding job.

We have developed TABSAT, a tool for the analysis of targeted bisulfite sequencing data generated on Ion Torrent systems. It was implemented using Python (version 2.7.6), R (version 3.1.2), and Perl (version 5.18.2) and makes use of several third party tools, including PRINSEQ [21], Bismark [19], and TMAP (version 4.4.8) to address different tasks of the analysis workflow (depicted in Fig 1). TABSAT is available as a standalone application (either as Docker image or as installable source code) and as an application in the web-based Platomics platform (see Fig 2 and section “Availability of TABSAT”). In addition to Ion Torrent data, TABSAT can be used with data from the Illumina MiSeq platform. The tool is capable of handling data generated by Bisulfite-Sequencing PCR (BSP) [22] as well as Methylation-specific PCR (MSP) [23].

Upon execution of TABSAT, data is gathered from the user-specified sources and processed by the application. Results are organized into folders and stored under the given output path (see section “Using TABSAT”). The first step of the pipeline performs quality assessment using the PRINSEQ [21] software. For each input file, summary statistics, including read length, GC content, quality score distributions, and the number of read duplicates, are created. These metrics along with graphics are summarized in HTML reports, which can be easily displayed in a web browser. After inspection of the quality results, the user can decide which TABSAT parameters (see section “Using TABSAT”) should be used for further downstream processing. By default low quality bases (phred score < 20) are clipped at the end of reads. In addition, filtering of reads based on a user defined length can be performed, which removes all reads that do not meet the minimum length requirement. After finishing the quality control step a second HTML report is generated, which allows users to compare the quality of data before and after quality assessment.

**Fig 3:**
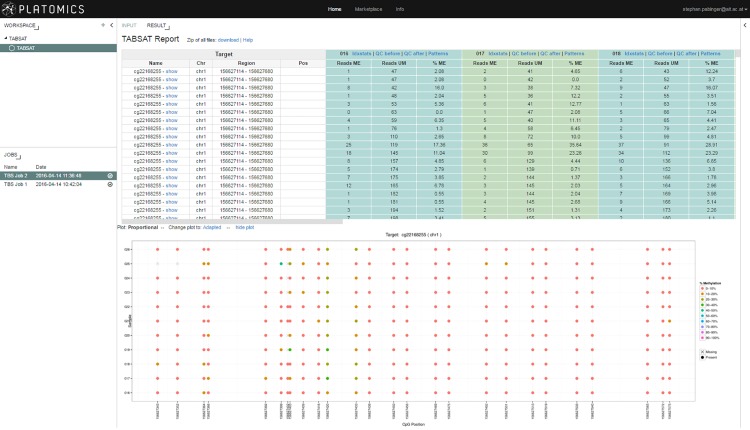
The result of a TABSAT run using the Platomics platform is shown. The output table of a run is presented in the upper part of the large panel containing the sequencing results for each CpG site of all samples. The lower panel shows the graphical representation of the methylation results. Additional information, such as mapping statistics and quality control information, about each sample can be accessed by clicking on the corresponding links.

The subsequent alignment module uses Bismark to map the reads to the defined reference genome (download links for human genome version hg19 and mm10 are provided at http://github.com/tadkeys/tabsat). In order to improve the performance of Bismark with Ion Torrent data, we have included the TMAP mapper into the program (see section “Modifications to Bismark”). The pipeline uses the *non-directional* Bismark setting as default, but can be changed to *directional* depending on the library used. Next, methylation levels are determined based on the previous mapping result. The final output table (see Fig 3) is generated using a BED [24] file, which is created during the methylation calling step. Alignment strand-specific as well as CpH information is available in additional output files.

**Fig 4:**
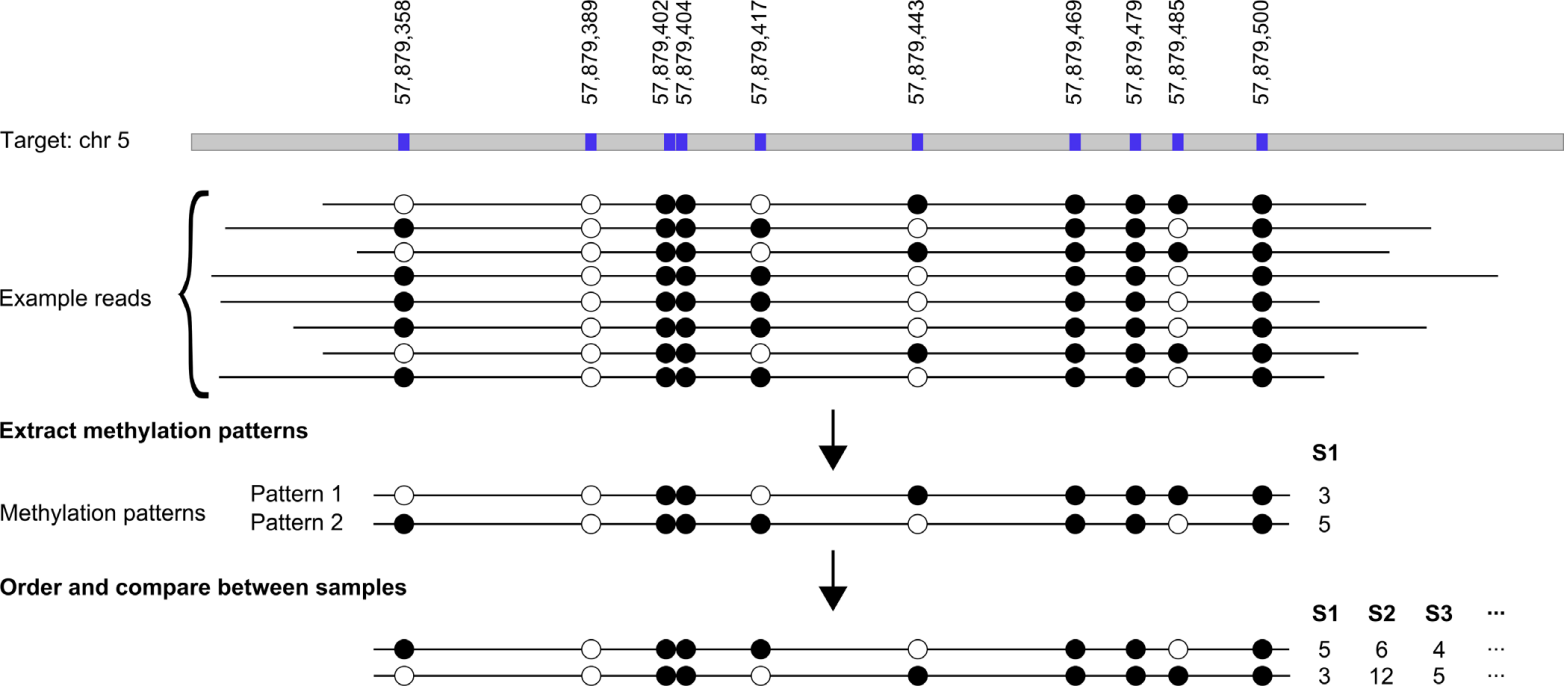
This image illustrates the determination and comparison of methylation patterns. The top line shows an example target region where the CpG coordinates are highlighted. Displayed below are several reads mapping to this target with different methylation patterns. From these reads methylation-patterns are extracted, ranked according to their frequency, and the patterns are compared between samples.

Due to the PGM’s ability to produce reads with a length of up to 400 base pairs, entire PCR amplicons can be covered by single reads. Consequently, it is possible to determine the methylation pattern across a single amplicon by taking the information from reads spanning the whole amplicon target (see Fig 4). For example, an amplicon containing 3 CpG sites has 8 (2^3^) different methylation patterns, ranging from fully unmethylated to fully methylated. For each sample and for each target the first step is the selection of all reads, which completely cover the target. Based on a user adjustable value, the user can select the percentage of the target that needs to be covered by a single read. Next, the different methylation-patterns are extracted, aggregated, and ranked based on their occurrence. In a final step, the patterns of all samples are compared with each other and their respective frequency is reported in a text document.

The output creation module aggregates the generated methylation information of all individual targets for all samples into a final table (see Fig 3). Several filtering steps are applied to remove false methylation calls (see section Using TABSAT): first low coverage methylation calls are removed from the output using a cutoff filter that can be set as a parameter. In addition, TABSAT determines the average coverage for each CpG per target/sample using the methylated and unmethylated coverage information. Next, it calculates a mean from these averages for each sample/target combination, which is used to remove every methylation call where the total coverage of a CpG is lower than the calculated mean coverage minus one standard deviation. As we use an amplicon sequencing approach, we expect a uniform coverage for each sample/target. By taking the average coverage of all CpGs within one sample/target and comparing it to the sum of each CpG we ensure to remove only extreme outliers. In addition, all CpGs that are not on the user specified strand are filtered out.

**Fig 5:**
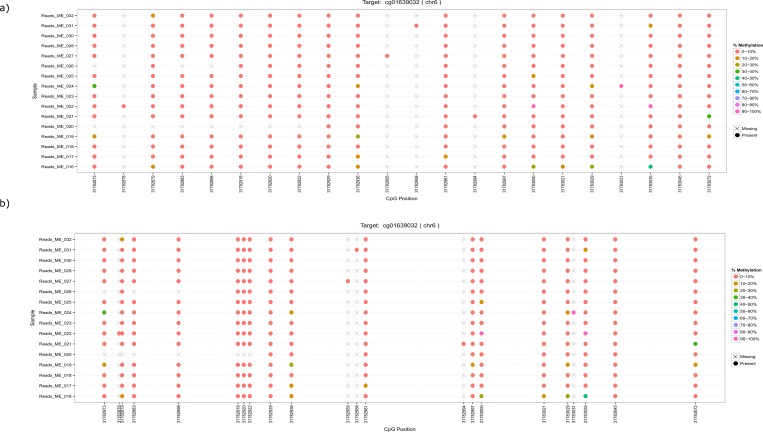
Displayed is an example of the two automatically generated lollipop diagrams containing the CpGs of the target region: a) all CpGs equally spaced across the target; b) all CpGs spaced according to their true chromosomal coordinate.

Finally, for each sample and for each CpG in the target region the number of methylated reads, the number of unmethylated reads, and the corresponding methylation percentages are reported. The results are also visualized as lollipop diagrams (see Fig 5) providing an intuitive way to investigate the methylation of the CpGs. Depending on the size of the target regions, it might be the case that all CpGs of the target region cannot be adequately displayed if they are spaced according to their position in the target region. Therefore, two versions of the lollipop diagram are created: a) CpGs are equally spaced along the target; b) CpGs are spaced according to their actual chromosomal coordinate. In addition to the graphical representations, general statistics, such as mapping results, and target coverage are reported.

### Availability of TABSAT

TABSAT is open-source and freely available. The pipeline consists of several modules including quality assessment, genomic alignment, methylation calling, CpG filtering, merging of samples, as well as methylation pattern calculation, visualization, and summarization of results. The pipeline is available in three versions: i) as Docker container with configured dependencies, required software, human genome assembly hg19, and mouse genome mm10 (available at https://github.com/tadkeys/tabsat) ii) as source code to be installed on a local infrastructure (accessible at https://github.com/tadkeys/tabsat) and iii) as a Platomics (http://demo.platomics.com) web application.

The Platomics platform supports creation, execution, and distribution of life-science applications. It manages storing, retrieving, organizing, and analysis of data and provides an interface to create life-science applications. A configurable output dashboard enables analysis results to be presented according to the needs of the application. The Platomics TABSAT version can be freely accessed at http://demo.platomics.com and after successful login (credentials available on github page) the user can perform analyses by selecting TABSAT in the workspace panel on the left side. After setting the correct parameters, target, and input files, the analysis is started by clicking on the “Start Job” button (see Fig 2). Currently running analyses are displayed in the lower left panel and results can be viewed by clicking on the corresponding job (see Fig 3). The complete result set can be downloaded as a compressed file allowing further downstream analyses.

The user manual including information for the setup of the pipeline using the source code as well as the Docker container can be found at https://github.com/tadkeys/tabsat. Our pipeline has no limitations concerning the number or length of FASTQ sequences and the used reference genome assembly. Test datasets can be downloaded at https://github.com/tadkeys/tabsat. They include FASTQ files and target files.

### Modifications to Bismark

Bismark is a software package containing routines to map reads to a reference genome (using Bowtie [25], or alternatively Bowtie 2 [26]) and determine their methylation state. It provides an attractive combination of processing speed, genomic coverage, and quantitative accuracy and is one of the most widely used tools to map and analyze bisulfite treated short reads [17,18,27]. The software is able to handle single-end as well as paired-end reads of both directional and non-directional bisulfite libraries. Bismark is open-source, written in Perl and executed from the command line. Due to the initial poor alignment results (see Discussion) and the specific error profile of reads produced by the Ion Torrent systems, we decided to replace Bismark’s default mapper Bowtie2 (2.2.4) with TMAP (4.4.8) [20], the read mapping program provided by the Ion Torrent suite. TMAP has been specifically optimized to handle reads from Ion Torrent sequencing platforms in order to meet specific data mapping challenges. We have therefore extended the source code of Bismark to support the use of TMAP as an additional mapping program. Specifically, a new method to create the index of the reference genome and to perform single-end (SE) alignment using TMAP was integrated.

### Performance Evaluation

We processed the sequencing data of 48 samples (as described in the methods section) using the standalone version of TABSAT on a Linux Ubuntu 14.04 server with 16 cores and 32 GB memory. Using the Ion Torrent “314” sequencing chip, 16 barcoded samples (each having 53 amplicon targets) were multiplexed per run yielding on average approximately 25,000 reads per sample (~380 reads per amplicon) with an average length of 125 base pairs. In total 1,543,767 reads were analyzed, where 1,196,822 reads could be aligned to the human reference genome (hg19) yielding a mapping average of 77.9% and a mean target coverage of 282X. Results of all 48 samples were aggregated and reported in a final table.

### Mapping performance

To assess the performance of the improved mapping capabilities, we used one of the three targeted bisulfite datasets (314 chip) with 16 samples (each with 53 targets) to compare the output of the modified Bismark-TMAP version to the default version of Bismark (0.13.1). Hereby, we could observe that the average number of mapped reads per dataset increased from 31,469 to 54,395 (45.23% to 78.12%). Consequently, the average coverage of the targets improved from 362X to 483X. In addition, we could observe an increase of the average coverage of CpG sites corresponding with the 450k chip (see below) from 392X to 599X (see Table 1). It should be noted that bisulfite conversion introduces additional homopolymer stretches, which are traditionally difficult to analyze on Ion Torrent systems. However, TABSAT analyses only CpG sites, and therefore avoids these homopolymer rich regions.

**Table 1:** Output comparison using 16 datasets between the standard version and the Ion Torrent optimized version of Bismark. Parameters standard version: -bowtie2—non_directional; parameters Ion Torrent version: -tmap—non_directional.

	Bismark–default	Bismark—TMAP
Total # of reads (16 datasets–each with 53 targets)	1,114,979	1,114,979
Mapping average	45.2%	78.1%
Average number of mapped reads per sample	31,469	54,395
Coverage average	362X	483X
Coverage average of 450k targets	392X	599X

### Correlation with Illumina Infinium HumanMethylation450

**Fig 6:**
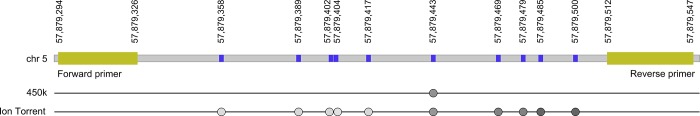
An example target region with marked CpG positions and forward/reverse primers is shown. The 450k target is depicted below as well as the CpGs measured using targeted sequencing. Grey color intensity is correlated to the methylation signal (450k) and percent methylation (sequencing).

In order to assess the performance of the targeted bisulfite sequencing setup, we compared the obtained sequencing results to an Illumina 450k methylation chip experiment using the same 48 DNA samples as used for targeted bisulfite sequencing (see Fig 6).

**Fig 7:**
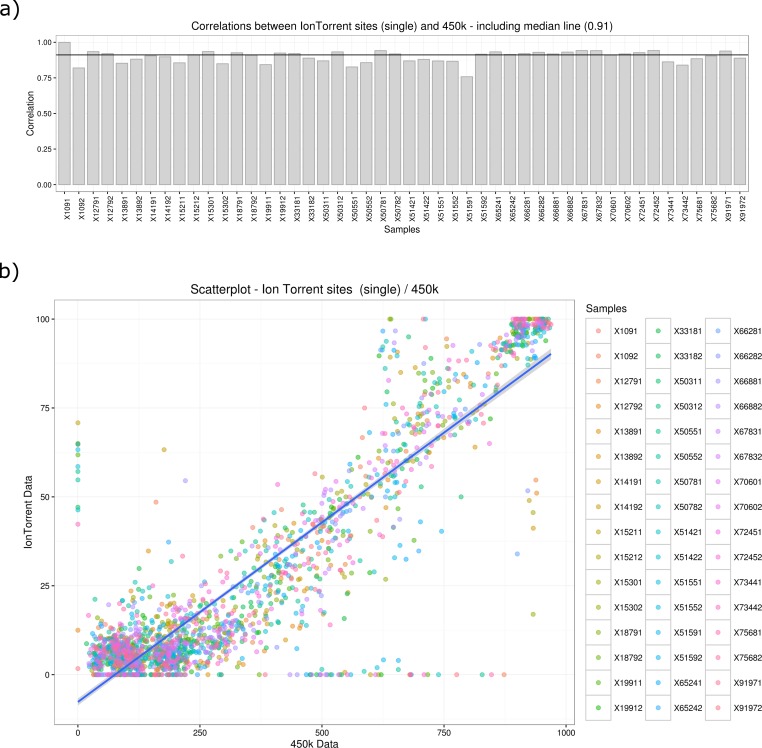
Presented is the correlation result between the Illumina 450k methylation chip and targeted bisulfite sequencing data: a) Barplot of the correlation for each sample; b) Scatterplot of 450k (x-axis) versus targeted bisulfite data (y-axis).

We correlated the methylation results of single Illumina 450k probes with the sequencing result of the corresponding single CpG for 53 targets per sample (see Fig 7).

Details about the selected amplicons, such as position, length, number of CpGs, melting temperature, GC percentage and number of mapped reads, are presented in Table 2 and S1 Table. The calculated median Pearson correlation yields 0.91 with a median R2 of 0.83. We also calculated the correlation between the average methylation of each target, instead of the single corresponding CpG, to the 450k result yielding a median correlation of 0.89 with a median R2 of 0.79.

**Table 2:** Details of the used amplicons: position on the human reference genome hg19, 450k methylation site ID, length of the target, number of CpGs, melting temperature, GC percentage, and average number of mapped reads (per sample).

Oligo_ID	Chr	Start	End	Target_ID	Length	#CpGs	Tm	%GC	Avg reads
BSP_1	chr1	156627114	156627680	cg22168255	567	35	84.05	62.08%	79
BSP_2	chr1	169555680	169556264	cg16054275	585	11	81.20	55.04%	475
BSP_3	chr2	236401705	236402272	cg04856858	568	38	83.29	60.21%	259
BSP_4	chr4	56685682	56686292	cg16606320	611	17	79.61	51.06%	2120
BSP_5	chr4	123842961	123843567	cg11169569	607	29	80.01	52.06%	180
BSP_6	chr5	92956435	92957014	cg07803236	580	50	84.85	63.97%	220
BSP_7	chr5	102898348	102898928	cg07655627	581	23	80.29	52.84%	71
BSP_8	chr5	140811383	140811992	cg07489502	610	33	80.18	52.46%	28
BSP_9	chr6	31627378	31627976	cg15415945	599	10	81.45	55.59%	284
BSP_10	chr6	31782716	31783294	cg01639032	579	30	83.83	61.49%	4
BSP_11	chr6	69344106	69344700	cg14031178	595	21	78.15	47.56%	12
BSP_12	chr6	166578845	166579411	cg20114732	567	15	78.05	47.44%	83
BSP_13	chr7	5013232	5013795	cg20160885	564	39	83.09	59.75%	58
BSP_14	chr7	8476807	8477405	cg06981279	599	17	76.93	44.57%	158
BSP_15	chr9	139939384	139939993	cg10664754	610	28	84.68	63.44%	192
BSP_16	chr10	6620279	6620895	cg01389728	617	12	76.96	44.57%	79
BSP_17	chr10	50886735	50887303	cg05378118	569	14	77.05	44.99%	382
BSP_18	chr10	135054458	135055053	cg13493048	596	21	81.14	54.87%	205
BSP_19	chr11	66206007	66206593	cg10495424	587	32	83.57	60.82%	348
BSP_20	chr12	56521723	56522328	cg12620342	606	32	84.85	63.86%	227
BSP_21	chr13	78271193	78271766	cg07847733	574	25	82.75	58.89%	113
BSP_22	chr13	95952581	95953201	cg26685941	621	21	79.75	51.37%	258
BSP_23	chr17	17400283	17400878	cg22981296	596	25	83.48	60.57%	191
BSP_24	chr17	26699283	26699850	cg23887396	568	43	85.16	64.79%	1670
BSP_25	chr17	77136522	77137125	cg17042243	604	12	81.27	55.13%	184
BSP_26	chr17	80372192	80372799	cg07929164	608	25	79.10	49.84%	250
BSP_27	chr18	74820183	74820764	cg16762684	582	6	76.02	42.44%	506
BSP_28	chr19	13213177	13213768	cg15013019	592	37	87.11	69.43%	573
BSP_29	chr19	18319085	18319669	cg15234155	585	26	82.25	57.61%	95
BSP_30	chr19	52390719	52391331	cg10635122	613	45	82.49	58.08%	1800
BSP_31	chr19	55863991	55864613	cg26562532	623	26	83.90	61.48%	140
BSP_32	chr19	58038315	58038921	cg10729426	607	43	80.01	52.06%	439
BSP_33	chr19	58514467	58515036	cg12259537	570	32	80.61	53.68%	135
BSP_34	chr20	36156592	36157203	cg19650416	612	23	80.05	52.12%	266
BSP_35	chr21	46360144	46360741	cg23285465	598	41	81.21	55.02%	432
BSP_36	chrX	69509900	69510480	cg16256230	581	31	82.40	58.00%	386
BSP_37	chr14	64854967	64855580	cg00691969	614	31	82.52	58.14%	187
BSP_38	chr10	436294	436908	cg01361261	615	32	80.68	53.66%	464
BSP_39	chr11	43755257	43755859	cg02468154	603	18	79.33	50.41%	84
BSP_40	chr16	15188232	15188848	cg03152385	617	25	80.41	53.00%	477
BSP_41	chr6	15538245	15538860	cg04611912	616	5	80.38	52.92%	516
BSP_42	chr3	55515356	55515967	cg05781968	612	31	84.00	61.76%	730
BSP_43	chr11	1469829	1470436	cg07479615	608	36	87.13	69.41%	218
BSP_44	chr19	35617292	35617893	cg08480266	602	44	88.01	71.59%	1434
BSP_45	chr5	180288564	180289135	cg11635304	572	28	80.83	54.20%	179
BSP_46	chr5	139682567	139683184	cg11924368	618	22	78.92	49.35%	437
BSP_47	chr10	27703028	27703636	cg15283904	609	27	81.63	55.99%	433
BSP_48	chr7	4797688	4798279	cg17466748	592	24	80.87	54.22%	217
BSP_49	chr8	145531650	145532255	cg22989161	606	19	81.33	55.28%	263
BSP_50	chr11	47236289	47236904	cg26408937	616	34	84.24	62.34%	1116
BSP_51	chr6	138893354	138893947	cg00224202	594	18	79.49	50.84%	119
BSP_52	chr6	33048458	33049047	cg12893780	590	37	85.11	64.58%	565
BSP_53	chr11	111169587	111170180	cg15081566	594	58	87.84	71.21%	220

## Discussion

We have developed a novel tool for the analysis of targeted bisulfite sequencing, which is especially equipped to handle, in addition to Illumina data, sequences generated on Ion Torrent systems. To date, several tools exist for the analysis of bisulfite data (reviewed here [17]), amplicons from bisulfite flowgram sequencing (Amplikyzer [28]) and locus-specific analysis of 5-methylcytosine (BiQ Analyzer HiMod [29]). However, none of these tools is specifically tailored for the analysis of Ion Torrent sequencing data and provides a one-stop solution from raw sequencing data to final results. Furthermore, the Ion Torrent PGM software platform currently does not support the analysis of bisulfite sequencing data. TABSAT comprises an analysis pipeline containing quality control, alignment, methylation calling, and output generation. In order to select the best mapping software for our purpose, we have evaluated several bisulfite analysis programs, such as Bismark [19], BS-Seeker2 [30], and BSMAP [31]. All programs produced similar or poorer mapping results, and offer different downstream analysis capabilities. Preliminary analysis with one input file using default parameters resulted in around 45%, 42%, and 20% of mapped reads for Bismark, BsSeeker2, and BSMap, respectively (see https://github.com/tadkeys/tabsat/tree/master/comp_tools). Based on the availability of the source code, the possibility to integrate a different mapping program, and the positive reviews, we decided to include Bismark in our workflow to handle mapping of sequencing reads and methylation calling.

Due to the initial suboptimal alignment results of the default Bismark version, we decided to incorporate TMAP [20] into the Bismark software, a dedicated mapper for Ion Torrent reads. Reads from Ion Torrent sequencing devices are usually longer than their Illumina counterparts and show a distinct different error profile, especially in homopolymer regions. The boost in read length causes an increased number of sequencing errors per read, which requires changing the mapping settings as the standard parameters for controlling mapping error may be too strict. As the supported aligners in Bismark (bowtie or bowtie2) are configured to be used with Illumina sized reads, we decided to include the Ion Torrent TMAP program, which has been designed to overcome these limitations.

Consequentially, we could show that the number of mapped reads increased on average from 31,469 to 54,395 (45.2% to 78.1%) on an evaluation dataset including 16 samples. Therefore, users will be able to process more samples on a chip, reducing the cost per sample. In order to check for correct mapping, we compared the number of positions where reads mapped outside of the target regions between the default and the TMAP Bismark versions. We expect reads to map outside of the target region, for example, due to unspecific PCR amplification. On average, we observed 2.7 additional positions where reads mapped outside of the target region when running the TMAP version, which can be explained due to the increased mapping potential of the TMAP program.

TABSAT supports the analysis of multiple samples (e.g. all barcoded samples on one chip) at once and outputs a combined result table for all samples. This facilitates interpretation and comparison of multi-sample studies. An important issue when working with sequencing data is to remove false results introduced by sequencing errors. Therefore, we have included a filtering mechanism into the workflow to reduce the number of uncertain methylation calls based on read coverage and artifacts. This strategy allows the generation of reliable results from which to draw biological meanings. Another important part of data analysis is the intuitive visualization of results. We have incorporated an automatic graph generation procedure which outputs two different types of lollipop diagrams. In addition, results are reported in tabular format, which can be used in further downstream analysis methods.

Since the Ion Torrent PGM is capable of producing reads with a length of up to 400 base pairs, we can use them to extract specific methylation-patterns. As one read originates from one distinct biological source, it could be possible to deduce biological causes that lead to different methylation-patterns in one sample. Especially interesting is the comparison of methylation-patterns between different biological groups, which may contribute to new methods for group classification. Consequently, descriptive statistics based on these methylation-patterns are automatically calculated for each sample and subsequently compared between samples.

The whole analysis solution has been designed to work with minimal user input and outputs results in clearly arranged tables and lollipop diagrams. The large number of Ion Torrent PGM sequencers available world-wide shows that there is a large community, which would benefit from this tailored analysis of bisulfite sequencing data. In addition, more than 100 Ion Proton sequencers have been registered on omicsmaps.com, which generate reads with a similar error profile as the PGM. Consequently, they would also benefit from a dedicated analysis workflow for generating high-quality results. Therefore, the presented work will help to unlock the power of Ion Torrent (and potentially Ion Proton) for bisulfite sequencing and DNA methylation analysis.

TABSAT is designed for amplicon studies and does not support the analysis of whole genome bisulfite sequencing projects. Furthermore, it is not limited to, but works best with amplicons smaller than 500bp as these can be efficiently visualized using lollipop diagrams. The aim of TABSAT is to cover the primary analysis of raw targeted bisulfite sequencing data to obtain methylation information for each analyzed cite. Given the provided comprehensive output, researchers can use additional downstream tools, such as the R project for statistical computing, to compare the methylation level between different groups of samples.

To assess the accuracy of TABSAT, we compared the sequencing results with data from an Illumina 450k methylation chip. The calculated median correlation of 0.91 confirms that targeting bisulfite sequencing on an Ion Torrent PGM yields accurate and reproducible results. A recent publication [32] has demonstrated effective methylated DNA immunoprecipitation sequencing (MeDIP-Seq) [33] on an Ion Torrent PGM, which was successfully validated using a 450k chip. This study shows the practicability of performing DNA methylation sequencing studies on a PGM and emphasizes the need for dedicated analysis solutions.

TABSAT can be used as standalone software, conveniently available as a Docker container. Docker containers wrap up software in a complete filesystem that contains everything it needs to run, making it an ideal solution to execute bioinformatics software in a self-contained and precisely controlled environment [34]. In addition, TABSAT is available as an embedded application within the Platomics life-science data analysis system. This graphical, web-based user interface has been especially designed to be usable without informatics knowledge. The results are presented in an intuitive way enabling an exploration of the data. As the complete result set can be downloaded as a compressed file, further in-depth downstream analysis can be easily performed.

In summary, TABSAT offers a novel and unreported way to analyze and interpret targeted bisulfite sequencing data.

## Materials and Methods

### Sample Material

DNA samples from the TwinsUK cohort were provided by the Department of Twin Research at King’s College London (KCL), TwinsUk is the largest registry of adult twins in the United Kingdom. It started in 1992 and currently encompasses approximately 12,000 volunteer twins from all over the UK [35]. The study was approved by St. Thomas’ Hospital Research Ethics Committee, and all twins provided informed written consent to participate in the study. 24 monozygotic twin pairs (n = 48 samples) with a difference of 800 or more grams in birthweight between twins were selected for this study. DNA isolation was done from whole blood at the KCL’s laboratories and subsequently provided to AIT alongside sample annotation

### Illumina Infinium HumanMethylation450 BeadChip

gDNA isolated from peripheral blood of 24 MZTs were subjected to DNA methylation analyses using the Illumina Infinium HumanMethylation450 BeadChip (450k Chip) following the manufacturer’s protocol. Briefly, 500 ng DNA per sample was deaminated using the Zymo EZ DNA Methylation Kit. The contained bisulfite solution converted unmethylated cytosines into uracil, by removing the amino group of the cytosine, while 5’-methylated cytosines remained unaffected. Incubation was done by applying 16 cycles of a two-step temperature program, starting at 95°C for 30 seconds followed by 50°C for 60 minutes.

The deaminated DNA was eluted in 12 μl elution buffer of which 4 μl were subjected to the 450k protocol. That protocol combines a genome wide amplification, an enzyme based targeted fragmentation, and a cleanup of the DNA prior to hybridization onto the bead chips. Since one 450k chip allows the investigation of 12 samples, the 48 samples were randomized before hybridization on four 450k chips. The methylation module of Illumina’s Genome Studio Software was used to subtract the background from the raw data and to perform an Illumina specific normalization which depends on internal control probes. Received beta-values were exported to a text-file for further analysis using R (V2.15) and the IMA package [36].

### Targeted bisulfite sequencing

Fifty-three differentially methylated loci (DML) identified by the 450k chip were selected for further analyses by targeted bisulfite sequencing using the Ion Torrent PGM platform (see S1 Table). MSRE-HTPrimer [37] was used to design high quality assays with a length of 150–320 bp for these 53 DML. BSP assays to amplify the methylated and unmethylated alleles were designed to cover the region of each DML probe of the 450k chip. Consequently, each individual assay contains the CpG of interest and multiple additional CpGs (typically up to 30). The designed BSP assays were setup and subsequently used to enrich the respective regions in the same 48 samples used for the 450k analysis. Target enrichment was done by qPCR single reaction, followed by pooling together all 53 targets for each sample. Library preparation and targeted sequencing on the PGM was conducted according to the manufacturer protocol. In brief, an individual barcode and a sequencing adapter were attached to the pooled targets (53) of each sample. Next, a pool containing all targets and all barcoded samples (48 samples each with 53 targets) was prepared for the final sequencing protocol, which includes an emulsion PCR to enrich the targets on the microspheres. The 48 samples were split into 3 equal batches of 16 samples. Each batch was loaded onto an Ion 314 chip and analyzed on the PGM.

### Using TABSAT

In order to run the command line version of TABSAT, a system with a Linux operating system and current hardware (50 GB HDD, 16GB RAM) is required. Installation descriptions are detailed at http://github.com/tadkeys/tabsat. The Platomics version of TABSAT can be freely used and does not require any installation. In order to try-out the software please consult the information at https://github.com/tadkeys/tabsat/blob/master/demo.md. TABSAT can be used with the following parameters:

**Target file [-t]:** The first input is the target file containing the region of interest. The pipeline accepts a tab-separated text file containing name, chromosome, start position, end position, and strand for all targets.

**Library [-l]:** This input can be either directional (DIR) or non-directional (NONDIR) depending on the used bisulfite sequencing adapters.

**Genome [-g]:** This parameter can be either hg19 (human) or mm10 (mouse).

**Sequencing library [-e]:** This input can be either SE (single end) or PE (paired-end).

**Aligner [-a] (optional)**: This input selects the aligner (TMAP or Bowtie2) used for mapping the reads to the reference genome.

**Minimum read length [-m] (optional):** This parameter is used for filtering reads that are shorter than the given threshold.

**Minimum 3’ read quality [-q] (optional)**: Bases that are below the given threshold are removed from the 3’ end of the reads.

**Percent of target covered by a read for pattern creation [-p] (optional):** This value specifies the percentage of the target that needs to be covered by a read to include it for pattern analysis.

**Minimum number of mapped reads per CpG [-r] (optional):** Number of reads that need to be present at each CpG site.

Lollipop sort order [-s] **(optional):** List of samples that is used to specify the order in the lollipop plots.

**Output directory [-o]:** This parameter determines where the output of the analysis run is stored.

**Input files:** With the last input the user specifies a list of FASTQ files (one or many targeted deep bisulfite sequencing runs) as produced by the sequencer. If a barcoded library was used, FASTQ files need to be generated for all barcodes before use (barcode splitting is not performed by TABSAT). Optionally the user can specify a directory (using–d) containing several FASTQ files.

## Supporting Information

S1 Table This table contains information about the 53 used amplicon targets for bisulfite sequencing. (XLSX)
